# Rectus Sheath Hematoma in a Recovering Dengue Patient: A Case Report

**DOI:** 10.7759/cureus.49338

**Published:** 2023-11-24

**Authors:** Pragya Sinha, Virendra S Chauhan

**Affiliations:** 1 Radiodiagnosis, Rama Medical College and Research Institute, Hapur, IND; 2 Radiodiagnosis, Saral Diagnostics, Noida, IND

**Keywords:** affordable healthcare, health promotion in medical imaging, dengue virus infection, complications of dengue fever, dengue fever/complications

## Abstract

A 63-year-old male patient recovering from dengue came to our department for contrast-enhanced computed tomography (CECT) for the evaluation of abdominal pain. An ultrasound performed in the periphery diagnosed him with a rectus abscess. The CECT evaluation done in our department clarified that the collection in his rectus sheath was hemorrhagic and not infective, as previously thought. The patient was managed conservatively and recovered without complications.

Like most infectious diseases, dengue is a disease of tropical countries. System-wise data collection processes are inadequate in many developing countries, which means complications and adverse effects of common diseases are not adequately captured. Furthermore, resource limitations restrict the availability of more expensive diagnostic tests to central locations. Peripherally located regions with lower purchasing capacity have greater access to relatively inexpensive tests. This causes deficits in the management of some common disease entities, like dengue.

Considering these issues, it is important to optimize healthcare testing for low-resource settings. This can only be achieved with adequate sensitization of healthcare providers in diagnosis and management.

## Introduction

Febrile patients with severe musculoskeletal pain often go unrecognized in emergency departments due to inadequate sensitivity towards muscle sheath hematomas associated with the presentation of dengue fever. A patient who presents with a fever of unknown origin and an exudative muscle collection on ultrasound can be incorrectly diagnosed as having a musculoskeletal abscess.

Dengue is a disease of the tropics [1,2]. Hemorrhage is a known side effect of dengue fever, leading to the formation of hematomas [3]. However, the management of dengue patients often overlooks musculoskeletal hematomas, especially in those with pyrexia of unknown origin (PUO). Increased sensitivity to the presence of hematomas in fever cases related to dengue will improve the diagnosis of this complication on ultrasound, especially in low-resource settings.

We will present a case of rectus sheath hematoma in a PUO patient, diagnosed as an abscess in the periphery.

## Case presentation

A 63-year-old male subject recovering from dengue came to our department with a complaint of pain and tenderness in the right-sided anterior abdominal wall. The patient was febrile (101°F) at the time of contrast computed tomography (CT) evaluation in our center. He also had raised blood glucose levels (275 mg/dl) and a decreasing platelet count (56,000/ml) at the time of the scan. An ultrasound in the periphery revealed gall bladder wall edema, minimal ascites, and right pleural effusion. Furthermore, the ultrasound revealed a collection in the right rectus muscle. Considering the subject’s clinical symptoms of high fever with abdominal pain, they considered the exudative collection to favor an infective abscess. Prior medical history included uncontrolled diabetes and essential hypertension.

**Figure 1:**
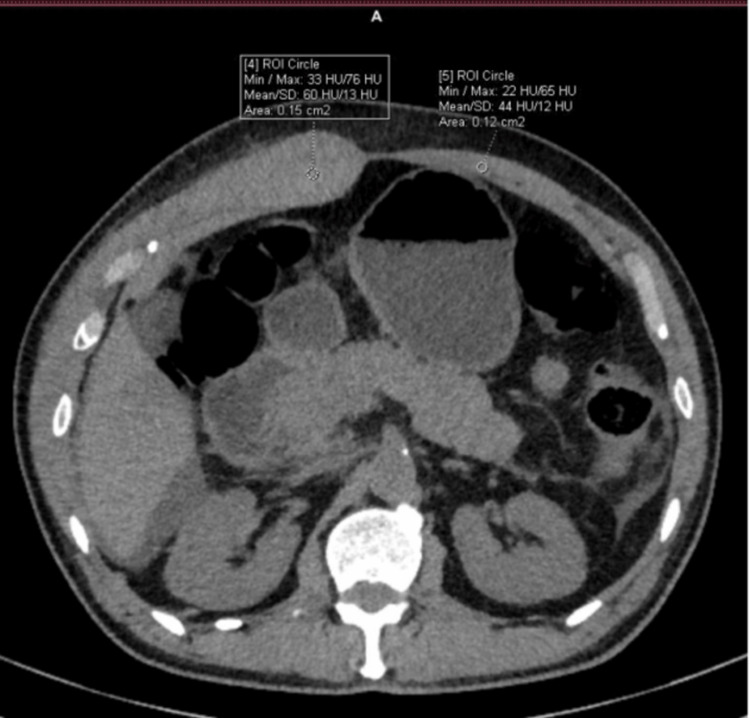
Non contrast CT image of subject showing abdominal wall CT: computed tomography

**Figure 2:**
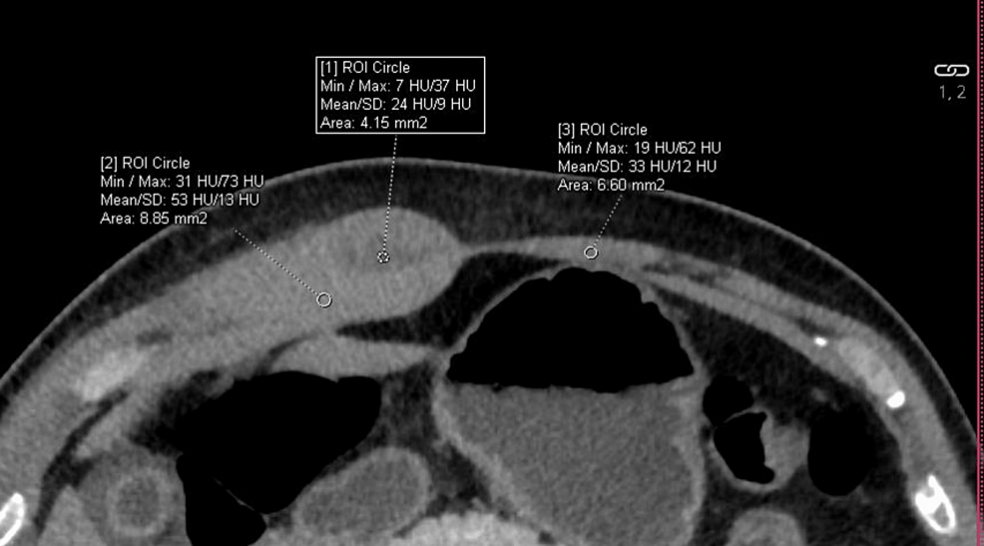
Right rectus sheath shows collection on CECT CECT: contrast-enhanced computed tomography

A CECT done in the department corroborated ultrasound findings of gall bladder wall edema and right pleural effusion. Minimal perihepatic-free fluid was also seen. In addition, the right rectus sheath appeared thickened (27mm vs. 7mm on the left) (Figure 1) and hyperdense (HU 60) (Figure 2) compared to the left rectus sheath (HU 44). Moreover, there was a non-enhancing hypodense collection within the muscle. Based on CECT, the patient was diagnosed as having rectus sheath hematoma, likely due to dengue coagulopathy.

The patient was managed conservatively with pain management and fluid replacement. The hematoma resolved in a few days, along with the resolution of symptoms.

## Discussion

Rectus sheath hematomas are very painful and sometimes form a palpable lump. They will often not cross the midline [2]. Ultrasound findings will mimic any exudative collection with echoes within the collection. However, thin septae and echogenic clots may suggest the diagnosis of hemorrhage. CT has a hyperdense pattern of intramuscular collection, with no enhancement in contrast. CT is the usual mode for a final confirmatory diagnosis of rectus hematoma.

A literature review on Pubmed yielded fourteen [3-16] articles regarding rectus muscle hematoma in dengue patients. Most of these were case reports. Muscle hematoma has since been recognized as an often occult cause of severe abdominal pain in dengue patients.

A total of ten case reports of abdominal wall hematomas in dengue patients were identified among the fourteen articles in the National Medical Library [4-13]. Nine of these were in the rectus sheath and one in the psoas muscle (n = 10). All these reports were from South Asia: India (n = 5), Sri Lanka (n = 2), Pakistan (n = 1), Indonesia (n = 1), and Singapore (n = 1). These locations represent the areas where dengue is more common and patients are more likely to suffer complications due to a lack of timely diagnosis. The mean age of the patients was 60, with 60% (n = 6) females and 40% (n = 4) males.

At least five of these patients were diagnosed with dengue only after the onset of abdominal pain (lower abdomen-3, upper abdomen-2) due to the hematoma. All five had been having PUO. The remaining five patients were being treated for or recovering from dengue and developed severe abdominal pain (lower abdomen-3, upper abdomen-2) caused by the hematoma.

Repetitive vomiting was the most common cause of precipitating hematoma formation (n = 4). Sixty percent of patients (n = 6) had lower abdominal pain. Three patients from the unrecognized febrile dengue group had co-morbid conditions of hypertension and diabetes. One had adenomyosis with accompanying anemia. One last patient was on warfarin post-surgery for aortic stenosis [12].

The final diagnosis was made based on CT-based investigations in the majority of these (non-contrast CT-4, CECT-1, CT angiography-1) cases.

Hematoma patients are managed conservatively with rest, fluid replacement, and pain relief. In contrast to this, abscesses need to be further managed with culture and sensitivity, fluid evaluation, and then antibiotics and drainage. Early recognition of a hematoma will spare the patients these unnecessary investigations and reduce the cost burden of the disease.

## Conclusions

Dengue is predominantly a disease in tropical countries. System-wise data collection processes are inadequate in many developing countries, causing inefficient data collection on associations, comorbidities, and complications of infectious diseases. Furthermore, resource limitations result in the concentration of more expensive diagnostic tests in central locations. Peripherally located regions with lower purchasing capacity have greater access to inexpensive tests. This causes deficits in the management of some common disease entities, like dengue. Considering these issues, it is important to optimize healthcare testing for low-resource settings. Achieving this requires adequately sensitizing healthcare providers.

In patients who have had an untreated viral pattern of fever (high-grade fever with muscle and joint pain and lymphocyte predominance on blood count), we suggest that abdominal pain and guarding should prompt a search for muscle hematoma formation. Musculoskeletal hematomas are easy to diagnose on ultrasound when there is a degree of suspicion for them. This is especially true for anterior wall/rectus sheath hematomas, which are easily visualized with superficial ultrasound probes. Increased sensitivity to the presence of musculoskeletal hematomas in PUO patients during dengue season will improve the diagnosis of this complication on ultrasound. This is especially true for settings where ultrasound is the main workhorse for diagnosis.
